# Performance and Prospects of [^68^Ga]Ga-FAPI PET/CT Scans in Lung Cancer

**DOI:** 10.3390/cancers14225566

**Published:** 2022-11-13

**Authors:** Paula E. Borgonje, Louise M. Andrews, Gerarda J. M. Herder, John M. H. de Klerk

**Affiliations:** 1 Department of Clinical Pharmacy, Meander Medical Center, Maatweg 3, 3813 TZ Amersfoort, The Netherlands; 2 Department of Pulmonology, Meander Medical Center, Maatweg 3, 3813 TZ Amersfoort, The Netherlands; 3 Department of Nuclear Medicine, Meander Medical Center, Maatweg 3, 3813 TZ Amersfoort, The Netherlands

**Keywords:** fibroblast activation protein, CAF, FAPI, lung cancer, PET/CT, [^68^Ga]Ga-FAPI

## Abstract

**Simple Summary:**

The purpose of this review was to provide an overview of all the available evidence regarding the use of fibroblast activation protein inhibitor (FAPI) as a tracer for positron emission tomography (PET) in patients with lung cancer. In several studies and case reports, radioisotope-labelled FAPI PET/CT showed a strong diagnostic performance in lung cancer. In addition, the possible prognostic value of FAPI PET/CT in lung cancer is proposed.

**Abstract:**

Fibroblast activation protein (FAP) could be a promising target for tumor imaging and therapy, as it is expressed in >90% of epithelial cancers. A high level of FAP-expression might be associated with worse prognosis in several cancer types, including lung cancer. FAPI binds this protein and allows for labelling to Gallium-68, as well as several therapeutic radiopharmaceuticals. As FAP is only expressed at insignificant levels in adult normal tissue, FAPI provides a highly specific tumor-marker for many epithelial cancers. In this review, current information on the use of [^68^Ga]Ga-FAPI PET/CT in lung cancer is presented. [^68^Ga]Ga-FAPI shows a high uptake (standardized uptake value = SUV_max_) and tumor-to-background ratio (TBR) in primary lung cancer lesions, as well as in metastatic lesions of other tumor types located in the lung and in lung cancer metastases located throughout the body. Where a comparison was made to [^18^F]FDG PET/CT, [^68^Ga]Ga-FAPI showed a similar or higher SUV_max_ and TBR. In brain and bone metastases, [^68^Ga]Ga-FAPI PET/CT outperformed [^18^F]FDG PET/CT. In addition to this strong diagnostic performance, a possible prognostic value of [^68^Ga]Ga-FAPI PET/CT in lung cancer is proposed.

## 1. Introduction

Lung cancer is the leading cause of cancer-related death in Europe, accounting for over twenty percent of all cancer-related deaths [1]. Histologically, lung cancer is classified into small-cell lung cancer (SCLC), non-small-cell lung cancer (NSCLC), pulmonary neuro-endocrine neoplasms (NENs) and “others”, where NSCLC is the most common [2]. 

Solid tumors such as these consist not only of malignant cells, but are situated in a Tumor Microenvironment (TME), which contains endogenous stromal cells (such as fibroblasts, mesenchymal cells, immune cells and vascular cells), held together by the extracellular matrix (ECM) [3]. This unique environment interacts with the malignant cells to promote tumor development, immune evasion, metastasis and therapeutic resistance [4]. A subpopulation of fibroblasts, called cancer-associated fibroblasts (CAFs), are involved in the growth, migration and progression of the tumor. These cells can be targeted by binding the fibroblast activation protein (FAP), a membrane-bound glycoprotein belonging to the dipeptidyl peptidase 4 (DPP4) family. Normal tissues have low and generally undetectable levels of FAP expression. A high expression of FAP occurs in wound-healing and inflammation, such as arthritis, atherosclerotic plaques and fibrosis, as well as in ischemic heart tissue, and in more than 90% of epithelial carcinomas [5,6]. As a result of these characteristics, FAP poses a promising target for the characterization of cancers. In several cancer types, high FAP expression is related to poor prognosis, indicating a possible predictive value [6,7,8,9]. 

**Figure 1:**
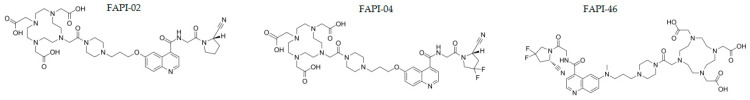
Chemical structure of FAPI subtypes used in clinical practice [11].

Furthermore, several radiolabeled FAP inhibitors (FAPI) targeting FAP expression in CAFs and characterized by the rapid renal clearance and high tumor-to-background ratio (TBR) have been developed for PET imaging. The derivatives FAPI-02, FAPI-04 and FAPI-46 allow for labelling with the radionuclide Gallium-68 [10]. The chemical structures of these FAPI subtypes are provided in Figure 1. 

In clinical practice, [^18^F]FDG PET performs well in the diagnosis and staging of lung cancer [12]; however, it is non-specific and may be associated with false positives, especially due to uptake in infectious lung diseases (specificity for FDG-PET performance in diagnosing lung cancer is 77% (95% CI, 73–80%) versus 61% (95% CI, 49–72%) in areas with infectious lung disease [13]). Additionally, [^18^F]FDG uptake is variable among lung cancer histotypes and one of the most common organs for lung cancer metastases is the brain, which has high [^18^F]FDG uptake and, thus, little contrast [2,14]. Due to these limitations, it is useful to investigate promising radiotracers such as [^68^Ga]Ga-FAPI as a tool for diagnosis and staging in lung cancer. In several studies, [^68^Ga]Ga-FAPI PET imaging has demonstrated an equal or better performance compared to [^18^F]FDG for the detection of tumors and metastatic lesions. It is also used as a complement to [^18^F]FDG to improve tumor-delineation for planned surgery or radiotherapy [15,16,17,18,19,20,21]. 

In this review, we aim to explicate the evidence and limitations on the use of [^68^Ga]Ga-FAPI PET/CT scans in lung cancer. PubMed library was searched using combinations of the terms “FAPI” “lung” “cancer” “FDG” “malignancy” “oncology” and “PET”. Articles were selected based on titles and abstracts, and via the references of other relevant articles. All articles that were found describing the use of [^68^Ga]Ga-FAPI PET/CT in lung cancer are listed in this review.

## 2. Prognostic Value of FAP-Expression

As FAPI binds to FAP in the tumor microenvironment, it can provide insight in the biological characteristics of tumors. As mentioned before, in several cancer types, high FAP expression is related to poor prognosis [6,7,8,9].

In a meta-analysis of fifteen studies, including a total of 1998 patients, Liu et al. describe eleven solid tumor types: colorectal cancer, pancreatic adenocarcinoma, non-small cell lung cancer, esophageal cancer, gastric cancer, ovarian carcinoma, breast cancer, medullary thyroid carcinoma, endometrial carcinoma, oral squamous cell carcinoma (SCC), and osteosarcoma. In the pooled analysis, high FAP expression was correlated with a greater risk of local tumor invasion, lymph node metastasis and distant metastasis, and with poor overall survival [22]. However, these results were not significant in all subgroups, and more specific analysis is needed to describe the prognostic value of FAP expression in lung cancer.

### 2.1. Lung Cancer

In the aforementioned meta-analysis, one study on 59 patients with NSCLC was included by Liao et al. They performed immunohistochemical staining with anti-FAP antibodies on specimens of resected tumors, and assessed the percentage of both staining and intensity. They showed no significant correlation between FAP expression and clinicopathological variables, including TNM stage, but overall survival was significantly better in patients with FAP-negative tumors (*p* = 0.027). Within FAP-positive tumors, a higher percentage of staining (*p* = 0.0087), as well as a higher intensity of staining (*p* = 0.05), were predictive of a shorter overall survival [23].

Later, Kilvaer et al. evaluated FAP expression in tumor specimens from 536 stage I-III NSCLC patients. They assessed the percentage of staining and determined the optimal cut-off point discriminating high/low expression groups according to survival. Contrary to Liao et al., they found no significant difference in overall survival between the high and low FAP-expression groups in the overall cohort (*p* = 0.07) or the adenocarcinoma subgroup (*p* = 0.986), and even a positive effect of high FAP expression on overall survival in the SCC subgroup (*p* = 0.043) [24]. They hypothesize that FAP-expressing CAFs interact with tumor cells and other players in the TME in differential ways according to the context, tumor stage and tissue of origin.

In an attempt to further clarify the relevance of FAP-expressing CAFs as a predictor for disease progression, Chen et al. used FAP-staining on 122 stage I–III SCC tumor samples and used this to grade the tissues as CAF-rich or CAF-poor. They demonstrated worse 3-year overall survival (HR 2.031, 95% CI 1.034–3.989, *p* = 0.040) and a worse 3-year disease-free survival (HR 1.987, 95% CI 1.019–3.874, *p* = 0.044) in the CAF-rich group. Furthermore, they suggest a close relationship between CAF density and both lymphangiogenesis and tumor angiogenesis, based on an increased lymphatic vessel density and microvessel density in the CAF-rich group compared to the CAF-poor group (*p* = 0.001 and *p* = 0.002) [25].

In 75 cases of lung adenocarcinoma, Shi et al. discriminated between FAP-expression in stromal cells and in the cytoplasm of tumor cells. They showed that a high FAP-expression in stromal cells was associated with a lower overall survival (*p* = 0.019), while FAP-expression in tumor cytoplasm had no significant effect on overall survival (*p* = 0.4) [26].

In summary, three of these four studies correlate high levels of FAP-expression with poor prognosis, whereas one study [24] associates high FAP-expression with better overall survival. The origin of this discrepancy remains unclear. However, there are differences in sampling, staining, grading and analysis between the studies, and none of the studies report what therapy the patients received, while this could be a relevant determinant of prognosis. More research on the prognostic value of FAP-expression in lung cancer is needed.

### 2.2. Prognostic Value of [^68^Ga]Ga-FAPI Uptake in PET

As described, there are implications that FAP-expression could be used as a prognostic marker in several cancer types, including lung cancer. In the studies mentioned above, FAP-expression was determined by immunohistochemical staining of the tumor tissue. For this technique, a specimen of the tumor is characterized in vitro by labelling a discriminative (colored) molecule to a specific target, in this case FAP. An advantage of this technique is that it allows for evaluation of cellular localization and staining patterns in the context of tumor structures. However, the results are affected by variable pre-analytical handling of the specimen, such as delay in fixation or inappropriate fixation time, as well as analytic variables, such as concentration of the antibody or incubation condition, and inter- and intra-observer variabilities. Moreover, this technique is relatively labor-intensive and requires either a biopsy or resection of the tumor [27].

Mona et al. confirmed a positive correlation between FAP tissue expression measured using immunohistochemistry scores and [^68^Ga]Ga-FAPI-46 uptake in PET/CT in 141 patients with fourteen types of solid cancers (SUV_max_: r = 0.781 (95%CI 0.376–0.936)) [28]. In lung cancer specifically, Wei et al. showed a strong positive correlation between [^18^F]F-FAPI-04-uptake and immunohistochemically determined FAP-expression in six surgical specimens (r = 0.938, *p* = 0.005) [29].

These results indicate that uptake in FAPI PET/CT could be used instead of immunohistochemical staining when assessing the prognostic value of FAP-expression in cancer. This has already been demonstrated in gastric cancer by Rong et al. In 21 patients, they performed [^68^Ga]Ga-FAPI-04 PET/CT before treatment and concluded that [^68^Ga]Ga-FAPI-04 uptake was significantly higher in patients not responding to immune checkpoint blockage therapy [30].

## 3. FAPI Imaging in Lung Cancer

Imaging of FAP-expression by [^68^Ga]Ga-FAPI PET/CT could provide insight in tumor characteristics because FAP-expression in adult normal tissue is low and generally undetectable. [^68^Ga]Ga-FAPI PET/CT can, therefore, be used in the diagnosis and staging of cancer [15,16,17,18,19,20,21]. In the upcoming paragraphs, the available evidence on the use of [^68^Ga]Ga-FAPI PET/CT for diagnosis and staging of lung cancer is presented.

### 3.1. FAP-Expression in Lung Cancer

Chen et al. demonstrated that FAP is widely expressed in lung cancer, especially in SCC (100%) and adenocarcinoma (85.7%). In twelve early adenocarcinoma cases, only three lesions were positive for [^18^F]FDG on PET/CT, while ten lesions were immunohistochemically positive for FAP [31]. This indicates a potential advantage for [^68^Ga]Ga-FAPI PET/CT over [^18^F]FDG PET/CT. In the following part, currently available research on [^68^Ga]Ga-FAPI PET/CT in lung cancer is presented.

### 3.2. Imaging of Primary Tumor Lesions

Only a few studies on FAPI PET/CT in lung cancer have been published. In two studies conducted at the University Hospital Heidelberg in Germany, [^68^Ga]Ga-FAPI was studied in multiple cancer types, including lung cancer. In the same center, a specific study targeting patients with fibrotic interstitial lung diseases and lung cancer was conducted. Giesel et al. compared [^68^Ga]Ga-FAPI PET/CT to [^18^F]FDG PET/CT in 71 patients with different cancer types, of which nine patients had lung cancer. They used several FAPI subtypes (FAPI-02, FAPI-04, FAPI-46 and FAPI-74), but did not specify this per patient or cancer type. They showed very low [^68^Ga]Ga-FAPI-uptake in normal lung tissue, similar to [^18^F]FDG (SUV_mean_ 0.48 vs. 0.46, *p* = 0.056). The analysis of the uptake in primary tumors was not specified per cancer type, unfortunately. In eight patients with lung metastases, no significant difference between [^68^Ga]Ga-FAPI and [^18^F]FDG was found (SUV_max_ 6.68 vs. 11.48, *p* = 0.641) [32]. Combined with the similar uptake in normal lung tissue, this suggests that TBR in lung lesions would be similar between [^68^Ga]Ga-FAPI PET/CT and [^18^F]FDG PET/CT.

Their colleagues Kratochwil et al. evaluated [^68^Ga]Ga-FAPI-04 uptake in eighty patients with 28 different cancer types, and classified the SUV_max_ as low (SUV_max_ < 6), intermediate (SUV_max_ 6–12) and high (SUV_max_ > 12). Lung cancer (*n* = 25) showed the 6th highest [^68^Ga]Ga-FAPI-04 uptake and had a SUV_max_ >12. The average uptake in the background was SUV_max_ 1.6 for blood-pool and 1.4 for muscle, resulting in a TBR of >7.5 for lung cancer and other cancer types with high uptake. This was a combination of primary tumors and metastases of lung cancer, since patient numbers were not sufficient to analyze these separately [21]. Therefore, it cannot be concluded that primary tumor lesions in the lung show these high TBRs. However, based on the low FAPI-uptake in normal lung tissue that Giesel et al. found, sufficient TBRs are expected.

Röhrich et al. used [^68^Ga]Ga-FAPI-46 in PET imaging of fibrotic interstitial lung disease and lung cancer, and showed that both of these conditions can be imaged using this technique. Discrimination between lesions of interstitial lung disease and lung cancer was performed by a multidisciplinary team based on clinical presentation, radiologic pattern on CT and in eight of fifteen patients, on additional lung biopsy. FAPI-46 uptake in fibrosis lesions was comparable to tumor lesions. However, in fibrosis lesions, as well as in the background tissue, SUV_max_ and SUV_mean_ decreased over time faster than in tumor lesions. This resulted in increasing TBR for the tumor lesions at 10, 60 and 180 min after injection [33].

Wang et al. compared [^68^Ga]Ga-FAPI PET/CT to [^18^F]FDG PET/CT in 27 primary and recurrent lung cancer lesions and reported a mean SUV_max_ of 13.7 for [^68^Ga]Ga-FAPI (subtype not specified) and 10.4 for [^18^F]FDG (*p* = 0.02). This resulted in a slightly higher TBR for [^68^Ga]Ga-FAPI PET/CT (34.2 versus 25.9, *p* = 0.02) [14].

In Table 1, an overview of the currently available information on the performance of [^68^Ga]Ga-FAPI PET/CT in cancer lesions in lung tissue, as described in this paragraph and complemented by information from case reports that are depicted below, is provided.

### 3.3. Imaging of Metastatic Lesions

#### 3.3.1. Locoregional Metastasis

In 2018, Loktev et al. were the first to compare [^68^Ga]Ga-FAPI imaging with [^18^F]FDG in a patient with locally advanced lung adenocarcinoma and they a showed significantly higher uptake of [^68^Ga]Ga-FAPI-02 compared to [^18^F]FDG in metastatic lesions, as well as lower uptake in background tissue, leading to higher contrast. The primary tumor was also clearly visible, but a quantitative comparison to [^18^F]FDG was not reported [36]. After this, several case reports were published on the detection of metastatic lesions of lung cancer using [^68^Ga]Ga-FAPI PET.

#### 3.3.2. Cerebral Metastasis

In a case of a 64-year old woman presenting with persistent headache, vomiting and altered consciousness, [^68^Ga]Ga-FAPI PET/CT was performed to identify possible metastases after a lung nodule was discovered using [^18^F]FDG PET/CT. This showed the presence of brain metastases that could not be detected by [^18^F]FDG because of the high physiological uptake, but also a higher SUV in the primary nodule (7.64 vs. 6.03). FAPI subtype was not specified [34].

Giesel et al. also describe a case where cerebral metastasis of lung cancer was detected because of [^68^Ga]Ga-FAPI PET/CT. In this case, suspicion for brain lesions was low based on FDG PET-CT, where limited tumor mass, with only a small T2a primary tumor and one potential N2 lymph node, was shown, without evidence of bone or visceral metastasis. [^68^Ga]Ga-FAPI-04 PET/CT, however, showed two FAPI-04-positive lesions in the brain that were confirmed by contrast enhanced cerebral MRI [37].

Fu et al. describe a case of a woman with newly diagnosed lung cancer, presenting with progressive pain in the left hip after one month. [^18^F]FDG PET/CT showed bone destruction of the left hip with high FDG uptake, as well as multiple FDG-avid areas in the bones, lungs, liver and lymph nodes. A brain lesion was also visible, with an SUV_max_ of 5.9. In addition, high FDG uptake was observed in the muscles of the right hip and lower leg. [^68^Ga]Ga-FAPI PET/CT showed that FDG-avid lesions were also FAPI-avid (FAPI subtype not specified). No increased uptake in the muscles was observed, supporting the theory that this increased FDG-uptake was due to overuse of the right leg. Interestingly, the [^68^Ga]Ga-FAPI PET/CT scan revealed two lesions in the humeri that were not visible on the [^18^F]FDG PET/CT scan (no confirmation of malignancy was reported for these lesions). As expected, the brain lesion showed a higher contrast than in the [^18^F]FDG PET/CT, despite a lower SUV_max_ (2.3 vs. 5.9) [38].

These case reports show the value of [^68^Ga]Ga-FAPI PET/CT in the detection of cerebral metastases of lung cancer. However, in current clinical practice, a brain MRI is part of the standard care for these patient groups.

#### 3.3.3. Lymph Node Metastasis

Shang et al. provide an example of the added value of [^68^Ga]Ga-FAPI PET/CT in the detection of lymph metastasis, as they report a patient with SCLC was staged T1bN2M0 based on multiple FDG-avid lymph nodes using [^18^F]FDG PET/CT. As part of a clinical trial, a [^68^Ga]Ga-FAPI PET/CT was performed one week later (FAPI subtype not specified). This showed the known primary tumor, with a SUV_max_ of 7.7, versus 2.7 in the [^18^F]FDG-scan. However, no increased FAPI uptake was detected in the suspicious lymph nodes. Histology confirmed the absence of malignancy in these nodes and the revised TNM stage was T1bN0M0 [35].

#### 3.3.4. Cardiac Metastasis

Another case emphasizing the clinical significance of [^68^Ga]Ga-FAPI PET/CT is presented by Liu et al. In this case, [^68^Ga]Ga-FAPI PET/CT (FAPI subtype not specified) aided in diagnosing a mediastinum type lung cancer that could have been easily misdiagnosed, due to the unusual presentation. Additionally, [^68^Ga]Ga-FAPI PET/CT was well able to distinguish lesions in the mediastinum, while high [^18^F]FDG uptake in the heart is usually physiological, and thus an [^18^F]FDG PET/CT might not have revealed the cardiac lesions [39].

The added value of [^68^Ga]Ga-FAPI PET/CT in the detection of lung cancer metastases is also supported by the findings of Wang et al., who compared the performance of [^68^Ga]Ga-FAPI PET/CT with [^18^F]FDG in 34 patients with stage IV lung cancer [14]. In line with the reported cases, they found that [^68^Ga]Ga-FAPI (subtype not specified) had higher TBR than [^18^F]FDG in the detection of all types of metastatic lesions, with the largest advantage in brain lesions (TBR 314.4 vs. 1.0, *p* < 0.001) and bone lesions (TBR 31.3 vs. 5.0, *p* < 0.001). This is especially interesting considering that the nervous system (39%) and bones (34%) are the primary metastasis sites for lung cancer [40]. Together, this is 73% of lung cancer metastases in which [^68^Ga]Ga-FAPI outperforms [^18^F]FDG to a large extent.

In Table 2 an overview of the information available at present on the performance of [^68^Ga]Ga-FAPI PET/CT in the detection of lung cancer metastasis, as described above, is provided.

### 3.4. Limitations of [^68^Ga]Ga-FAPI PET/CT in Lung Cancer

As described previously, Röhrich et al. showed that interstitial lung diseases, as well as lung cancer, cause increased FAPI-uptake [33]. Apart from these diseases, other lung conditions can show increased FAPI-uptake as well. This complicates the diagnosis of lung cancer based on [^68^Ga]Ga-FAPI PET/CT. Tang et al. report a case of a patient presenting with chest pain and cough that was initially diagnosed with lung cancer based on [^18^F]FDG PET/CT, which showed a soft tissue mass with intense [^18^F]FDG activity. Lymph nodes in the mediastinum and left hilar region were enlarged, and some of these showed increased FDG-uptake, whereas others did not. [^68^Ga]Ga-FAPI-04 PET/CT showed intense uptake in the soft tissue mass, but no increased uptake in the enlarged lymph nodes. Biopsy of the mass and the lymph nodes showed inflammatory cell infiltration and fibroblast proliferation, but no signs of malignancy [42]. This demonstrates that organizing pneumonia, as well as interstitial lung disease, should be considered during the diagnosis of a cancer-like pulmonary mass with increased [^68^Ga]Ga-FAPI-uptake.

An important limitation of [^68^Ga]Ga-FAPI PET/CT in the detection of lung cancer metastasis is the increased FAPI-uptake in several non-malignant processes, as is substantiated by the following cases.

In 2020, Liu et al. reported a case where [^68^Ga]Ga-FAPI-04 PET/CT showed a higher uptake in lung adenocarcinoma than [^18^F]FDG PET/CT (SUV_max_ 3.3 vs. 2.3, TBR not reported). However, [^68^Ga]Ga-FAPI-04 PET/CT also showed two FAPI-avid lesions in the vertebral column that were not visible on [^18^F]FDG PET/CT. An MRI scan revealed no signs of malignancy in the spine and the lesions corresponded to degenerative osteophytes. No FAPI-04-uptake was observed in other osteophytes, indicating that the FAPI activity may be associated with the presence of fibroblasts in early osteophytes [43].

Similarly, Wu et al. described a case in which a pulmonary nodule was identified by CT-scan in a woman who had a car accident ten days earlier. [^18^F]FDG PET/CT showed increased uptake in the pulmonary nodule, as well as in several lymph nodules, and slight metabolic activity in the L4 vertebral body. [^68^Ga]Ga-FAPI PET/CT did not show increased uptake in the lymph nodes, but did show intense uptake in the L4 vertebral body (FAPI subtype not specified). A biopsy of the lung nodule confirmed invasive lung adenocarcinoma. However, the FDG-avid lymph nodes were diagnosed as inflammatory lesions and the vertebral body with intensive FAPI-uptake was concluded to be a fracture caused by the car accident [44].

This corresponds with other observations of increased FAPI-uptake in degenerative osteophytes, fractures and arthritis, possibly mimicking bone metastasis [20]. These cases highlight the importance of critical CT image evaluation to aid in the differentiation between true bone metastases and other conditions.

## 4. Conclusions and Future Perspective

Several studies have demonstrated that [^68^Ga]Ga-FAPI uptake in lung cancer is high, whereas uptake in healthy lung- and other tissue is low, resulting in sufficient TBR for application in imaging. Especially in the detection of metastasis, SUV_max_ and TBR were higher for [^68^Ga]Ga-FAPI PET/CT than for [^18^F]FDG PET/CT, indicating a potential added value for [^68^Ga]Ga-FAPI PET/CT in the staging of lung cancer. A limitation is the increased FAPI-uptake in several non-malignant processes, including organizing pneumonia and interstitial lung disease, which should be considered.

In addition, three studies associating high levels of FAP-expression with poor prognosis in lung cancer, and one study correlating high FAP-expression with better prognosis, specifically in SCC, were discussed. As [^68^Ga]Ga-FAPI PET uptake is correlated to FAP-expression, we suggest that the relationship between FAP-expression and prognosis should be investigated in future studies using [^68^Ga]Ga-FAPI PET/CT.

As described previously, Wei et al. demonstrated a correlation between histochemically determined FAP-expression in lung cancer and [^18^F]F-FAPI-04 uptake in PET/CT. They suggested that ^18^F-labeled FAPI-04-uptake might distinguish different pathological types of lung cancer. They studied thirty patients with lung adenocarcinoma, seventeen patients with SCC and fourteen patients with small cell lung cancer. [^18^F]-FAPI-04 uptake in both primary tumors and metastases of all three subtypes was extensive. They showed no difference in uptake of FAPI in the different pathological subtypes, but did show that FAPI-04 uptake in metastases from SCC were the highest, as measured by the so-called SUV_max_, i.e., maximum standard uptake value (SUV_max_ 10.41 ± 6.96), followed by ADC (SUV_max_ 7.03 ± 4.30) and the lowest in SCLC (SUV_max_ 4.94 ± 2.60) [29].

[^18^F]F-FAPI might have good potential for use in lung cancer, as well as [^68^Ga]Ga-FAPI, as demonstrated by Li et al., who performed a prospective study on the primary staging of lung adenocarcinoma in 34 patients using [^18^F]F-FAPI-42. They demonstrated similar uptake in 27 primary tumors (SUV_max_ 12.54 for [^18^F]F-FAPI-42 versus 12.22 for [^18^F]FDG, *p* = 0.754) and were able to detect more metastases using [^18^F]F-FAPI than [^18^F]FDG (554 lesions versus 464 lesions, *p* = 0.003). For brain lesions, [^18^F]F-FAPI outperformed [^18^F]FDG in both detection (34 versus 9 lesions, *p* = 0.002) and TBR (9.53 vs. 1.01, *p* < 0.0001). However, contrast-enhanced MRI was still superior to [^18^F]FAPI PET/CT, detecting 56 lesions (*p* = 0.002) [41]. Depending on the local situation, [^18^F]F-FAPI might be more accessible to some hospitals, since the slightly longer half-life of ^18^F (110 min versus 68 min for ^68^Ga) allows for it to be labelled by a commercial external party. We expect that [^18^F]F-FAPI PET/CT, as well as [^68^Ga]Ga-FAPI PET/CT, will play a role in the staging and evaluation of lung cancer in the future, although more research is needed.

Furthermore, FAPI can be labelled to therapeutic radionuclides, such as Lutetium-177, Copper-64, Yttrium-90 or Actinium-225 [45,46,47,48], offering potential as a theranostic compound [49]. No records of using FAPI labelled to therapeutic radionuclides in primary lung cancer were found. However, Ferdinandus et al. used [^90^Y]Y-FAPI-46 in nine patients with several progressive, advanced-stage solid tumors, which all had metastatic nodes in the lung. They observed a low rate of attributable adverse events and signs of tumor response [48]. Additionally, in a case of sarcoma with lung metastases, three cycles of combined [^90^Y]Y- and [^153^Sm]-FAPI-46 attained stable disease for eight months [50]. Combined with the strong performance of [^68^Ga]Ga-FAPI PET/CT in lung cancer diagnostics, this suggests good potential for the labelling of FAPI to therapeutic radiopharmaceuticals and its application in lung cancer therapy in the future.

## Figures and Tables

**Table 1:** Comparison of SUV_max_ and TBR for [^68^Ga]Ga-FAPI and [^18^F]FDG in cancer lesions in lung tissue. P = primary lung tumors, M = metastatic lesions located in the lung.

	*n*	P/M	SUV_max_ [^68^Ga]Ga-FAPI	SUV_max_ [^18^F]FDG	TBR [^68^Ga]Ga-FAPI	TBR [^18^F]FDG
Giesel et al. [32]	8	M	6.68	11.48	NR	NR
Kratochwil et al. [21]	25	P + M	>12	NR	>7.5	NR
Röhrich et al. [33]	15	P	>15 ^1^	NR	>3.5 ^1^	NR
Wang et al. [14]	27	P	13.7	10.4	34.2	25.9
Hao et al. [34]	1	P	7.64	6.03	NR	NR
Shang et al. [35]	1	P	7.7	2.7	NR	NR

^1^ Values read from graph.

**Table 2:** Overview of SUV_max_ and TBR of [^68^Ga]Ga-FAPI and [^18^F]FDG reported for lung cancer metastasis.

	*n*	Location of Metastasis	SUV_max_ [^68^Ga]Ga-FAPI	SUV_max_ [^18^F]FDG	TBR [^68^Ga]Ga-FAPI	TBR [^18^F]FDG
Fu et al. [38]	1	brain	2.3	5.9	higher	lower
Li et al. [41]	17	brain	1.56	7.34	9.53	1.01
Wang et al. [14]	256 ^1^	lymph	12.8	6.9	11.6	4.3
	255 ^1^	distant (total)	12.9	5.8	40.5	6.2
	23 ^1^	brain	9.0	7.4	314.4	1.0
	109 ^1^	bone	15.6	7.0	31.3	5.0
	66 ^1^	pleural	11.5	4.3	28.7	10.9
	31 ^1^	lung	4.0	2.9	10.1	7.3
	11 ^1^	liver	5.9	6.7	8.0	2.9
	12 ^1^	adrenal gland	15.5	8.0	17.6	5.8

^1^ number of lesions, not patients.
